# Adeno-associated virus serotype rh10 is a useful gene transfer vector for sensory nerves that innervate bone in immunodeficient mice

**DOI:** 10.1038/s41598-017-17393-z

**Published:** 2017-12-12

**Authors:** Sun H. Park, Matthew R. Eber, Shunsuke Tsuzuki, Mary E. Booker, Aaron G. Sunil, D. Brooke Widner, Renee A. Parker, Christopher M. Peters, Yusuke Shiozawa

**Affiliations:** 1 Department of Cancer biology and Wake Forest Baptist Comprehensive Cancer Center, Wake Forest School of Medicine, Winston-Salem, NC 27157 USA; 2 Wake Forest University, Winston-Salem, NC 27109 USA; 3 Department of Anesthesiology, Wake Forest School of Medicine, Winston-Salem, NC 27157 USA

## Abstract

Adeno-associated virus (AAV) is frequently used to manipulate gene expression in the sensory nervous system for the study of pain mechanisms. Although some serotypes of AAV are known to have nerve tropism, whether AAV can distribute to sensory nerves that innervate the bone or skeletal tissue has not been shown. This information is crucial, since bone pain, including cancer-induced bone pain, is an area of high importance in pain biology. In this study, we found that AAVrh10 transduces neurons in the spinal cord and dorsal root ganglia of immunodeficient mice with higher efficacy than AAV2, 5, 6, 8, and 9 when injected intrathecally. Additionally, AAVrh10 has tropism towards sensory neurons in skeletal tissue, such as bone marrow and periosteum, while it occasionally reaches the sensory nerve fibers in the mouse footpad. Moreover, AAVrh10 has higher tropic affinity to large myelinated and small peptidergic sensory neurons that innervate bone, compared to small non-peptidergic sensory neurons that rarely innervate bone. Taken together, these results suggest that AAVrh10 is a useful gene delivery vector to target the sensory nerves innervating bone. This finding may lead to a greater understanding of the molecular mechanisms of chronic bone pain and cancer-induced bone pain.

## Introduction

The peripheral ends of sensory nerves express various ion channels and receptors^1^. Despite extensive research characterizing the function of these proteins in nociceptive and sensory processing, there is still a lot that is unclear regarding specialized roles of unique ion channels and receptors in different types of tissue. To dissect the role of these proteins in pain signaling it is useful to modulate the level of candidate genes expressed in sensory neurons. Although lentivirus, adenovirus, herpes-simplex virus (HSV), and adeno-associated virus (AAV) have all been widely used to deliver genes or short hairpin RNA (shRNA) to the nervous system^2–4^, AAV is often considered the preferred and safest method^5,6^. Several key traits of AAV are largely responsible for this preference: (1) AAV can widely diffuse throughout tissues, since its genome is smaller than other viruses^7^, (2) AAV has the ability to induce robust gene expression in organs/tissues of interest, since high titer virus production and purification are easily achieved^8^, (3) Gene manipulation following a single injection of AAV can induce sustained gene expression for months to years in the targeted organs/tissues^9–11^, and (4) AAV is associated with only mild immune responses^12^.

An important but understudied area of pain biology is cancer-induced bone pain (CIBP). CIBP is the most common complication associated with bone metastatic disease, which occurs in 70–80% of the patients with advanced breast and prostate cancer, and 30–40% of patients with lung cancer^13,14^, resulting in a significant impairment of quality of life. Unfortunately, currently available treatments for CIBP, including opioids and non-steroidal anti-inflammatory drugs (NSAIDs), come with severe side effects as well as the risk of abuse and addiction^15–17^. Therefore, it is critical to understand the underlying mechanisms of CIBP in order to develop more effective and safer therapies. Previous studies in rodents demonstrate an extensive innervation of skeletal tissue including bone marrow, mineralized bone, and periosteum by sensory neurons^18–20^, and growing evidence has demonstrated that cancer growth in bone increases the sprouting of sensory and sympathetic nerves innervating bone which may contribute to CIBP^21–23^. However, the extent to which the interaction between bone metastatic cancer cells and nociceptive neurons influences CIBP or disease progression is still unknown.

To better define the molecular mechanisms of CIBP development, there is a great need for methods that allow gene delivery to the sensory nerves that innervate bone. Several serotypes of AAV have been identified with nerve tropic signatures, but it is still unknown which AAV serotype is capable of or efficiently targets sensory nerves that innervate bone. In this study, we performed intrathecal injections of several serotypes of AAV and found that AAVrh10 has tropism for sensory nerves that innervate the bones of immunodeficient mice. Immunodeficient mice were chosen for this study because of the widespread use of xenograft models in the study of human disease in mice. Although further studies are clearly warranted, the use of AAVrh10 may open new avenues in the understanding of the molecular mechanisms of CIBP.

## Results

### AAVrh10 has highest tropism to sensory nerves

**Figure 1:**
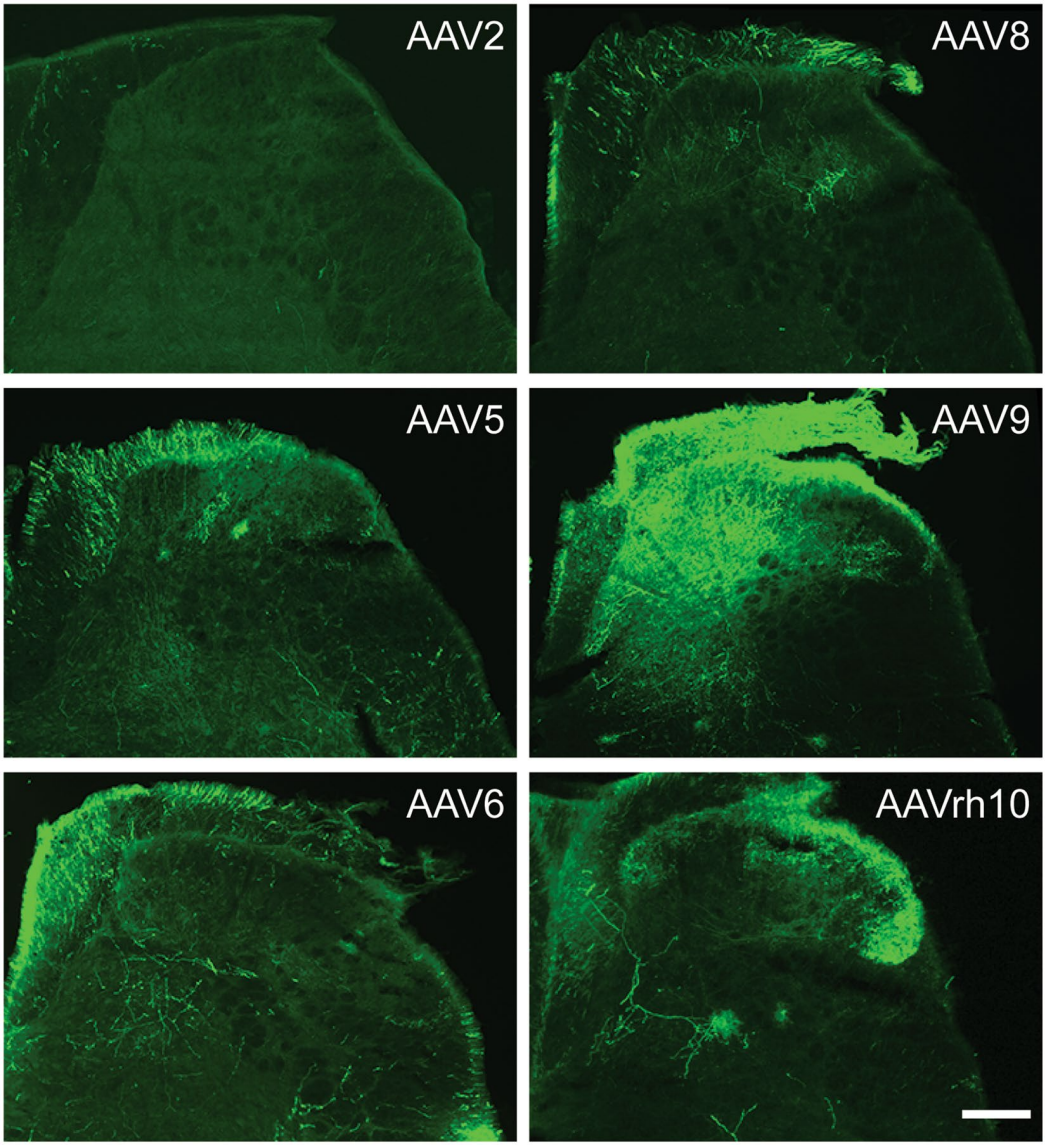
Distribution of AAV vectors in spinal cord. GFP-tagged AAV vectors (AAV2, 5, 6, 8, 9, and rh10) were placed intrathecally between the L4/5 vertebrae of immunodeficient mice by percutaneous lumbar puncture. At 4 weeks, spinal cords were collected for immunofluorescence analyses for GFP positivity to determine AAV distribution. Representative image of spinal cords obtained from AAV injected mice. Magnification, ×20. Scale Bar = 100 µm.

**Figure 2:**
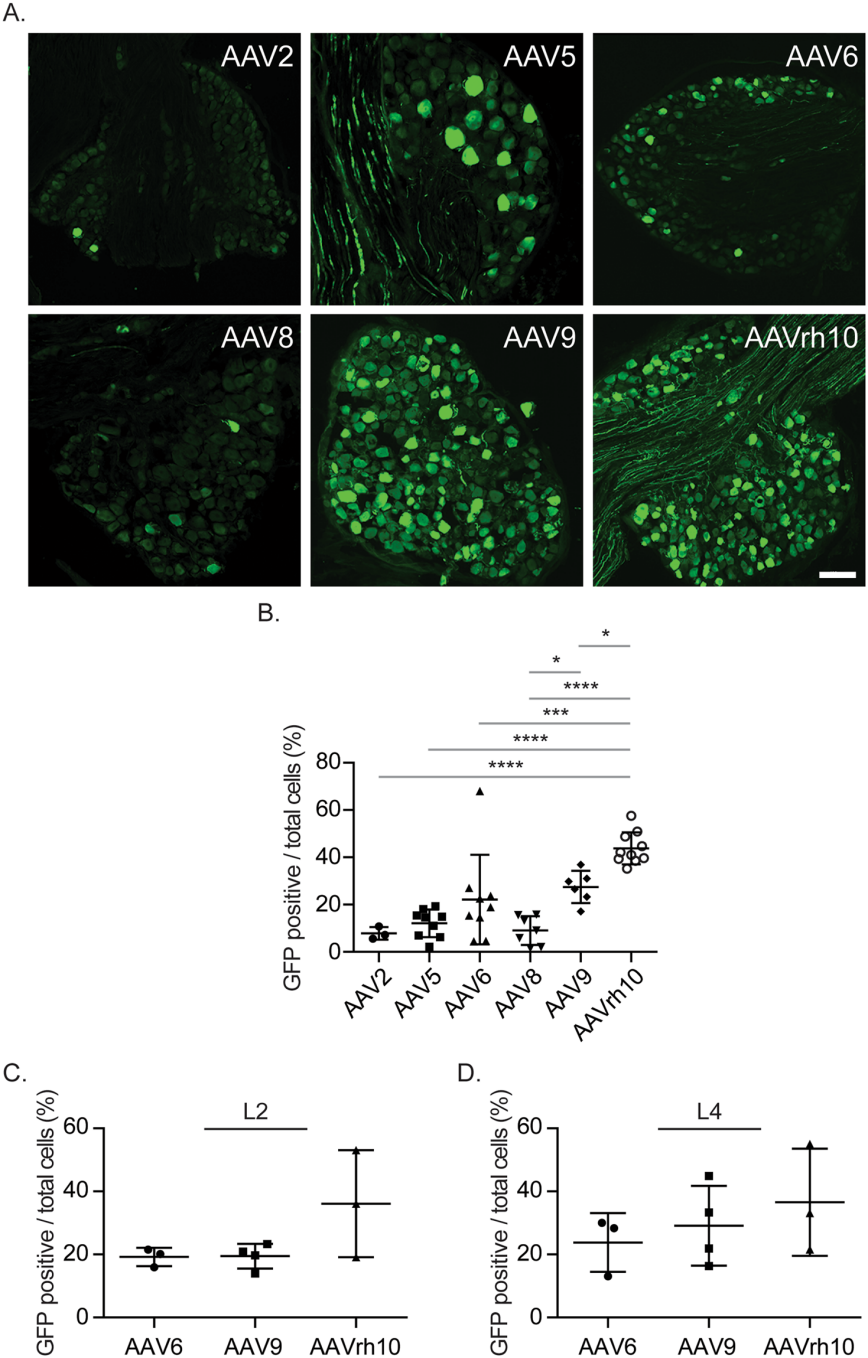
Distribution of AAV vectors in DRG. GFP-tagged AAV vectors (AAV2, 5, 6, 8, 9, and rh10) were placed intrathecally between the L4/5 vertebrae of immunodeficient mice by percutaneous lumbar puncture. At 4 weeks, DRGs were collected for immunofluorescence analyses for GFP positivity to determine AAV distribution. (**A**) Representative image of L3 DRGs obtained from AAV injected mice. Magnification, × 20. Scale Bar = 100 µm. (**B**) Quantifications of (**A**). (**C**,**D**) Quantifications of GFP positivity of (**C**) L2 and (**D**) L4 DRGs obtained from AAV injected mice. Presented as mean ± standard deviation, Significant differences were determined by one-way ANOVA with Tukey’s multiple comparisons. *p ≤ 0.05, ***p ≤ 0.0001, ****p ≤ 0.00001.

To determine which AAV serotype has superior gene transduction efficiency to sensory nerves innervating bones, we chose six different serotypes (AAV2, 5, 6, 8, 9, and rh10) which exhibit some degree of nerve tropism *in vivo* ^24–27^. Green fluorescent protein (GFP)-expressing AAVs were inoculated into severe combined immunodeficiency (SCID) mice by intrathecal injection, which is known to be an efficient delivery system to transfer viral vectors into the lumbar spinal cord and dorsal root ganglia (DRG)^25,28^. Four weeks after AAV inoculation, lumbar spinal cord and DRG were dissected for immunohistochemical analyses. Higher levels of transduction were observed in the dorsal horn of the spinal cord obtained from mice inoculated with AAV9 and rh10, whereas minimal or no virus transduction was detected in mice inoculated with AAV2, 5, 6, or 8 (Fig. 1A). Next, tropism to DRG was assessed in L3 DRGs. As was seen in the spinal cord, less efficient transduction was observed in the DRG obtained from mice inoculated with AAV2, 5, 6, or 8 (Fig. 2A and B). Interestingly, AAVrh10 showed significantly higher transduction efficiency in the DRG when compared to other serotypes, including AAV9 (Fig. 2B). Additionally, higher transduction efficiency was observed in the L2 and L4 DRGs obtained from mice inoculated with AAVrh10, compared to those from mice inoculated with AAV6 and 9, although there were no statistical differences (Fig. 2C and D). We therefore chose AAVrh10 for further analyses.

### AAVrh10 predominantly transduces myelinated and non-myelinated peptidergic sensory nerves

**Figure 3:**
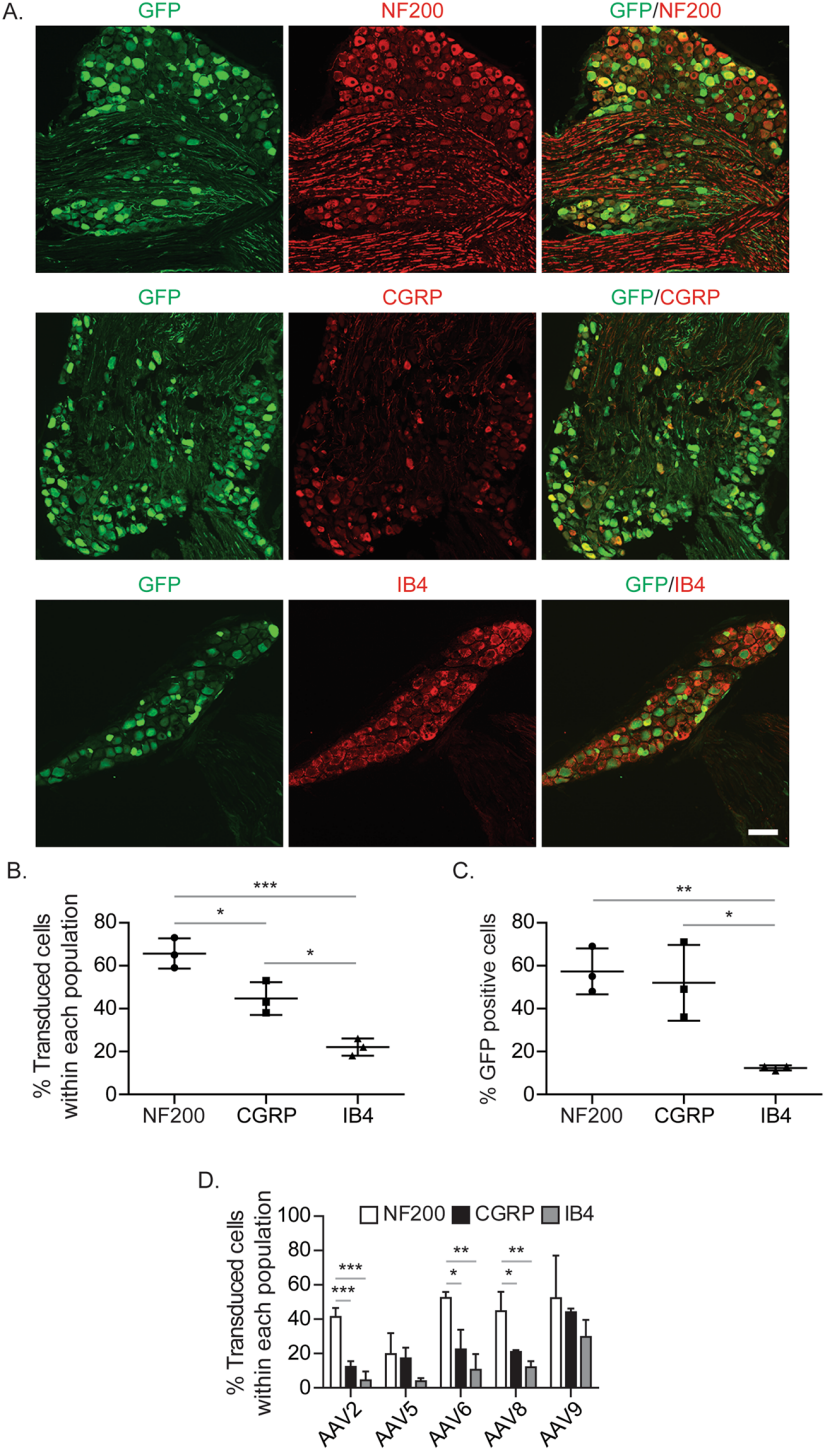
Subpopulations of sensory nerves in DRG transduced by AAVrh10. GFP-tagged AAVrh10 vector was placed intrathecally between the L4/5 vertebrae of immunodeficient mice by percutaneous lumbar puncture. At 4 weeks, L3 DRGs were collected for immunofluorescence analyses to verify expression in subpopulations of sensory neurons. (**A**) Representative image of co-localization of GFP positive cells, and myelinated neurofilament 200 (NF200), non-myelinated peptidergic calcitonin gene-related peptide (CGRP), or non-peptidergic isolectin-B4 (IB4) positive cells. Magnification, ×20. Scale Bar = 100 µm. (**B**) Quantification of the percentage of DRG cells transduced within different populations of sensory neurons (NF200, CGRP, or IB4). (**C**) Quantification of the percentage of total GFP positive DRG cells that co-expressed each marker. (**D**) Quantification of the percentage of DRG cells transduced within different populations of sensory neurons in the L3 DRGs obtained from AAV injected mice. Presented as mean ± standard deviation. Significant differences were determined by one-way ANOVA with Tukey’s multiple comparisons. *p ≤ 0.05, **p ≤ 0.001, ***p ≤ 0.0001.

**Figure 4:**
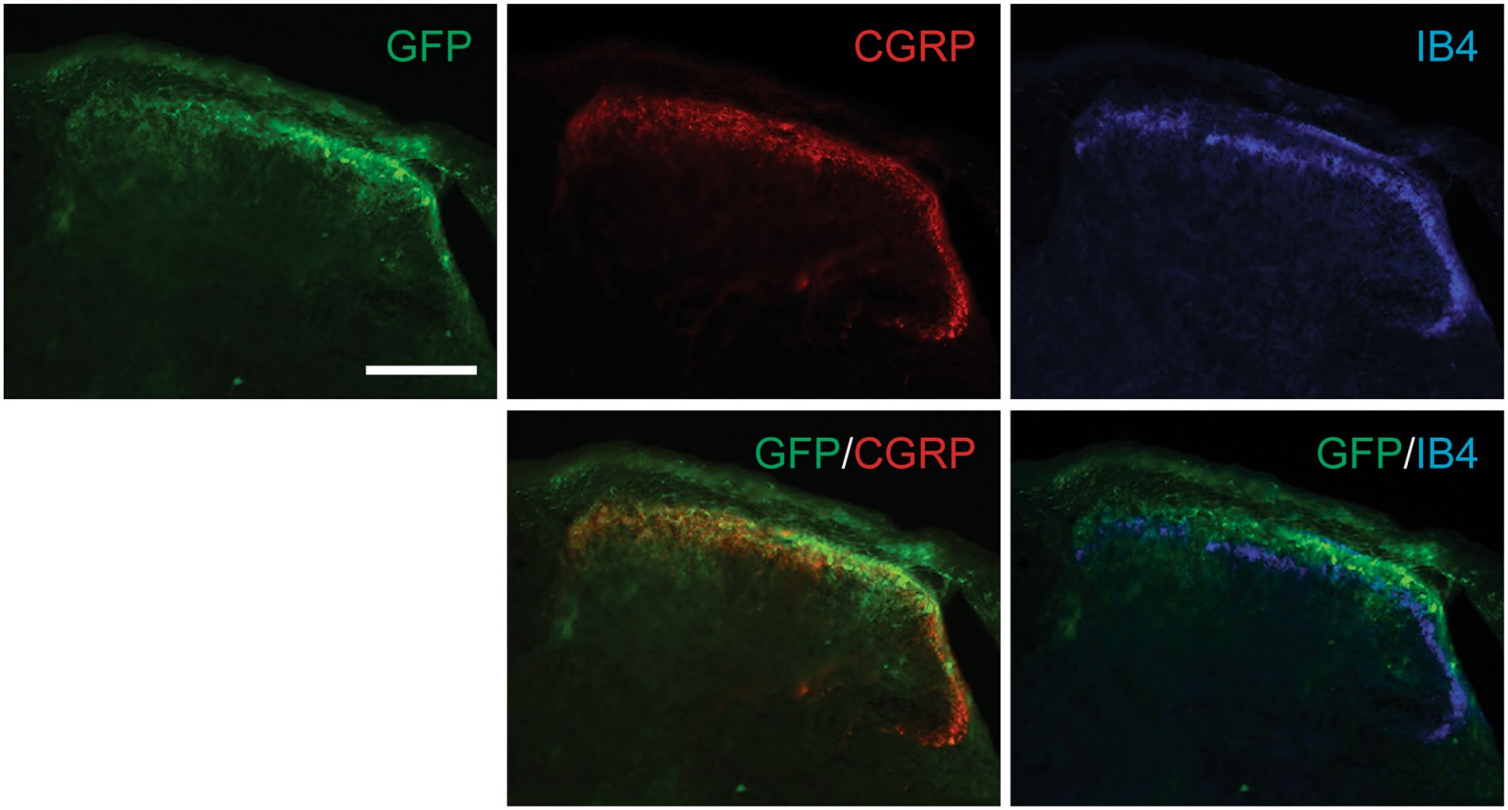
Subpopulations of sensory nerves in spinal cord transduced by AAVrh10. GFP-tagged AAVrh10 vector was placed intrathecally between the L4/5 vertebrae of immunodeficient mice by percutaneous lumbar puncture. At 4 weeks, spinal cords were collected for immunofluorescence analyses to verify expression in subpopulations of sensory neurons. Representative image of co-localization of GFP, CGRP, and IB4 in spinal cord. Magnification, ×20. Scale Bar = 100 µm.

It has been shown previously that the mouse femur is preferentially innervated by the myelinated (neurofilament 200 [NF200] positive) and non-myelinated peptidergic (calcitonin gene-related peptide [CGRP] positive) classes of sensory afferents, but not non-peptidergic (isolectin B4 [IB4] positive) sensory neurons^18,29^. To determine which subpopulation of sensory nerves are transduced by AAVrh10, the lumbar spinal cord and DRG were stained with NF200, CGRP, or IB4 (Fig. 3A). We quantified the percentage of sensory neurons that expressed GFP within each subpopulation (Fig. 3B) and the percentage of total GFP positive cells that co-expressed each marker (Fig. 3C). AAVrh10 demonstrated robust transduction of sensory neurons including both NF200 positive and CGRP positive sensory neurons, but significantly fewer IB4 positive sensory neurons. When we conducted subtype analysis following injection of other serotypes, although we saw a similar pattern of distribution (NF200 > CGRP > IB4), our decision to focus on AAVrh10 was affirmed by the fact that none of the other serotypes had both high transduction of NF200 (>60%) and CGRP (>40%) positive neurons, but significantly reduced transduction of IB4 (<25%) positive neurons (Fig. 3D). Within the spinal cord, GFP expression co-localized with CGRP positive afferent sensory neurons in the dorsal horn, and less with IB4 positive afferent sensory neurons (Fig. 4), similar to the DRG. Since NF200 is highly expressed through the entire spinal cord, we were not able to perform co-localization between NF200 and GFP specific to myelinated sensory nerves in the dorsal horn of spinal cord (Data not shown). These data suggest that AAVrh10 has specific tropism to the NF200 positive nerve fibers and CGRP positive nerve fibers.

### AAVrh10 transduces sensory nerve fibers that innervate bone

**Figure 5:**
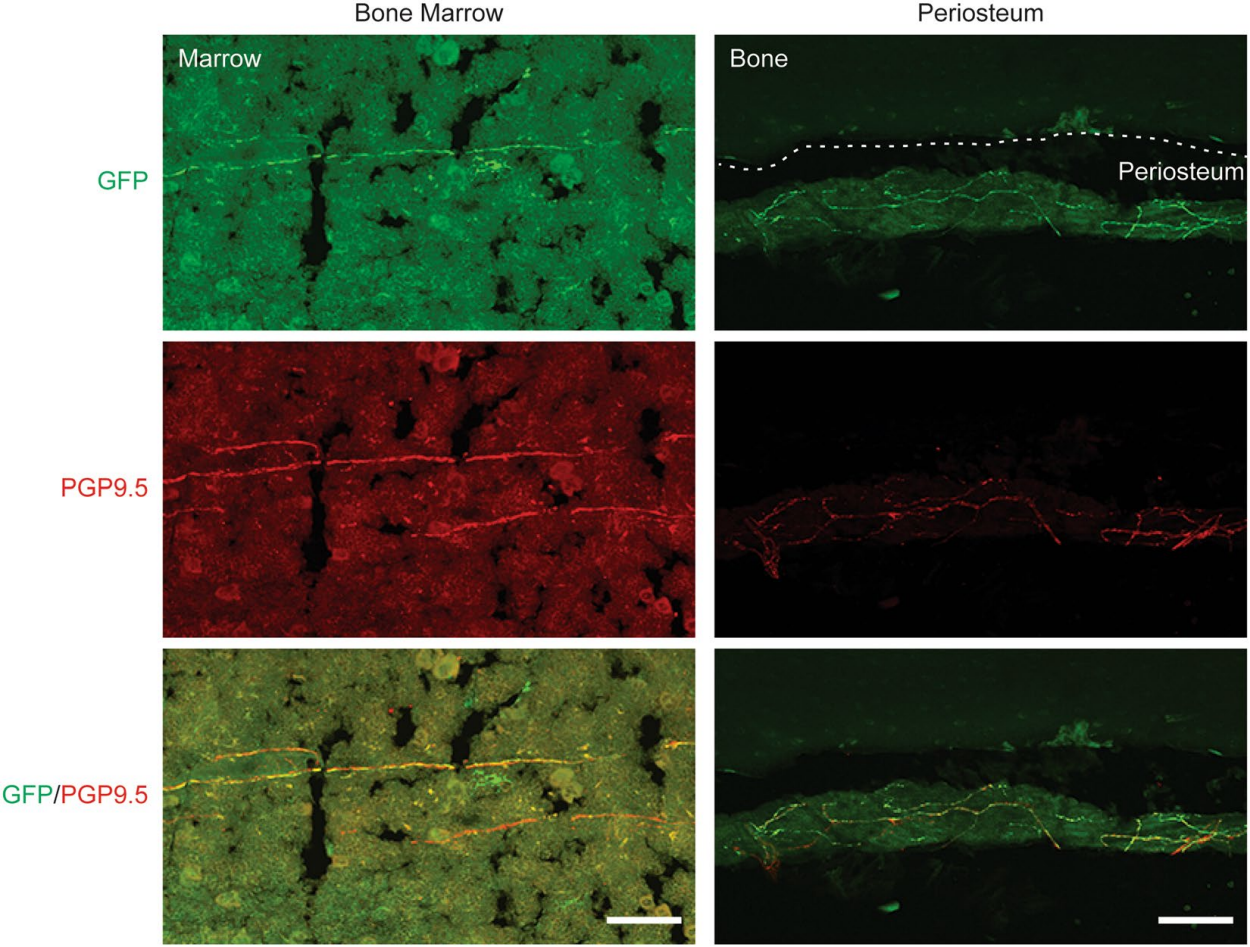
Distribution of AAVrh10 vector to sensory nerves in skeletal tissues. GFP-tagged AAVrh10 vector was placed intrathecally between the L4/5 vertebrae of immunodeficient mice by percutaneous lumbar puncture. At 4 weeks, femurs were collected for immunofluorescence analyses for GFP positivity to determine AAV distribution to sensory neurons. Representative image of co-localization of GFP and PGP9.5 in bone marrow and periosteum. Magnification, ×20. Scale Bar = 100 µm.

**Figure 6:**
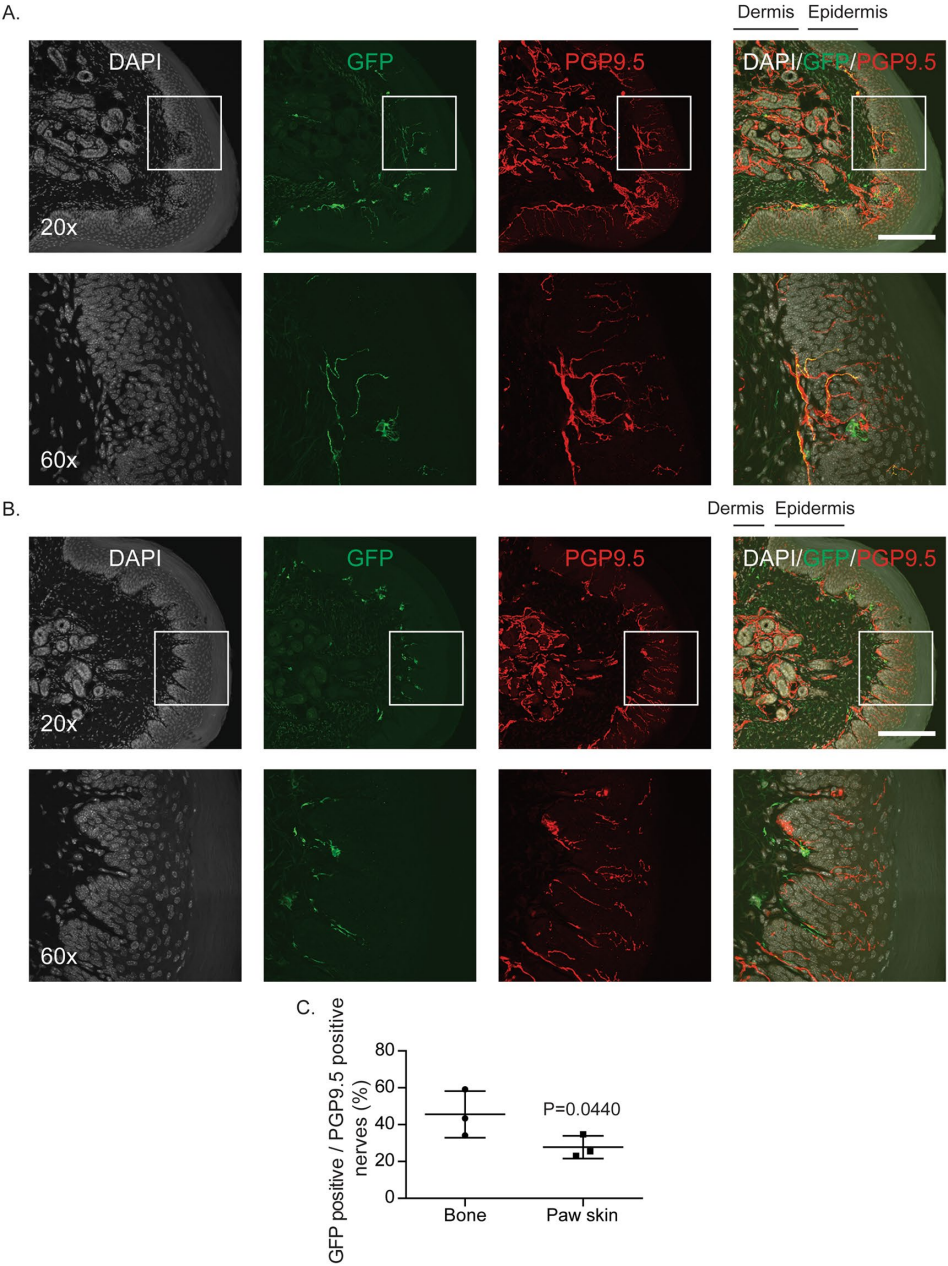
Distribution of AAVrh10 vector to sensory nerves in paw skin compared to those in skeletal tissue. GFP-tagged AAVrh10 vector was placed intrathecally between the L4/5 vertebrae of immunodeficient mice by percutaneous lumbar puncture. At 4 weeks, glabrous skin from the hindpaw was collected for immunofluorescence analyses for co-localization of GFP and PGP9.5 to determine AAV distribution in sensory neurons. (**A**) Representative images of GFP and PGP9.5 co-localization in paw skin sections. (**B**) Representative images of GFP and PGP9.5 without co-localization in paw skin sections. Scale Bar = 100 µm. (**C**) Quantifications of GFP positive sensory nerves over PGP9.5 positive sensory nerves between bones (Fig. 5) and paw skins (Fig. 6A and B) obtained from AAVrh10 injected mice. Presented as mean ± standard deviation, Significant differences were determined by Student’s *t-*test.

To investigate whether AAVrh10 can transduce genes in the peripheral sensory nerve fibers innervating bones, we assessed GFP immunoreactivity in femurs. GFP-expressing nerve fibers were observed in the bone marrow and periosteum (Fig. 5). Additionally, these GFP-expressing nerve fibers were co-localized with a specific marker for sensory neurons, PGP9.5 (Fig. 5). Finally, to examine how far AAVrh10 diffuses in peripheral nerve fibers, GFP expression was assessed in the paw skin. Most of the sensory nerve fibers in the paw skin were not transduced by AAVrh10, however GFP-expressing nerve fibers were occasionally observed (Fig. 6A and B), and some of them were co-localized with PGP9.5 positive sensory nerves (Fig. 6A). Analysis of the degree of co-localization between PGP 9.5 and GFP within each tissue revealed that a greater percentage of sensory nerves were transduced by AAVrh10 in the bone compared to the glabrous skin in the hindpaw (Fig. 6C). These data suggest that AAVrh10 can transduce the peripheral sensory nerves, and more predominantly those innervating bone.

## Discussion

In this study, we investigated tropism of AAV serotypes to sensory neurons innervating bone. GFP-expressing AAV vectors (AAV2, 5, 6, 8, 9, and rh10) were administrated to immunodeficient mice through intrathecal injection. We found that AAVrh10 transduces the sensory neurons in the DRG and spinal cord with higher efficiency than AAV2, 5, 6, 8, and 9. Additionally, AAVrh10 showed high tropism for the NF200 positive large myelinated and CGRP positive small peptidergic sensory neurons that innervate bone. Taken together, our findings suggest that AAVrh10 is a preferred serotype for delivering genes to sensory nerves innervating bone.

It has been appreciated that AAV9 and AAVrh10 have higher transduction efficiency for the central and peripheral nervous systems, compared to other serotypes of AAV^30–32^. Additionally, these two serotypes do not require mannitol pretreatments to enhance penetration of AAV vectors to the neurons, while others do^28,33^, further suggesting their higher affinity to central and peripheral neurons. We also found that transduction of these two vectors were more efficient in immunodeficient mouse spinal cords, when compared to AAV2, 5, 6, and 8. In DRG, there was significantly higher transduction efficiency in AAVrh10 (43.8%) than AAV9 (27.5%). Importantly, in addition to sensory neuron transduction, AAV9 is known to develop robust motor neuron transduction^34^, and it has been shown that AAVrh10 can also spread to motor neurons when injected intrathecally^35,36^. Likewise, in this current study, AAVrh10 occasionally transduced motor neurons in the spinal cord, but in those cases transduction efficiency was extremely low (data not shown). Therefore, these findings indicate that in our hands, AAVrh10 was the best AAV vector at targeting sensory nerves in this model.

We are aware that there may be concerns regarding the specificity of gene transduction of AAV vectors to sensory neurons. One strategy to address this issue is to develop sensory nerve specific gene-targeted animals using the Cre-flox system (e.g. crossbreeding between Advillin-Cre animals and animals with a floxed gene of interest)^37,38^. However, these models might cause off-target effects as changes in gene expression may occur in all sensory nerves. In terms of targeting specific types of sensory nerves (e.g. sensory nerves innervating bones), gene delivery systems using a viral vector may oftentimes be more useful. In fact, AAV delivery of Cre is a strategy that could circumvent this crucial issue of specificity. In this current study, we found that AAVrh10 has higher affinity to myelinated and small peptidergic sensory neurons, compared to small non-peptidergic sensory neurons. On the other hand, it has been shown that AAV9 transduces not only peptidergic sensory neurons, but also non-peptidergic sensory neurons^28^. Since the fibers that innervate bones are mainly myelinated and small peptidergic sensory neurons, and rarely non-peptidergic sensory neurons^18,29^, our findings suggest that AAVrh10 can be an alternative strategy for preferential targeting of sensory neurons in bone.

Bone pain is a major clinical problem in patients with bone metastases, and the complex interactions between bone metastatic cancer cells and sensory nerves make it difficult to treat. Gene therapy using AAV vectors are currently being tested in patients with neurodegenerative diseases, such as Alzheimer’s, Parkinson’s, and Canavan disease^39–41^. Subsequently, AAV gene therapy has been considered a potential alternative treatment for chronic pain without unwanted side effects. Moreover, AAVrh10 is being pursued as an attractive alternative to AAV2, which is the most frequently used serotype in clinical trial settings^42,43^. AAVrh10 was isolated from the rhesus monkey, while other serotypes including AAV2 were discovered in humans^11,44–46^. Therefore, neutralizing antibodies against AAVrh10 are less likely to be generated in human serum. Although further studies regarding its efficacy, safety, and long-term effects in a clinical setting are still warranted, a gene delivery system using AAVrh10 to target sensory nerves innervating bones may prove to be a better treatment strategy to improve the quality of life of cancer patients with bone metastases.

## Materials and Methods

### Adeno-associated virus (AAV)

The AAV2, 5, 6, 8, and 9 CMV-eGFP vectors generated using a Sf9/baculovirus system were purchased from the Iowa Viral Vector Core Facility of University of Iowa (Iowa City, IA). The AAVrh10 CMV-eGFP virus generated in HEK293 cells was purchased from the Penn Vector Core in the Gene Therapy Program of the University of Pennsylvania (Philadelphia, PA). The titer of each virus was within a range from 3 × 10^12^ to 6 × 10^13^ vector genome (VG)/ml (Table 1).

**Table 1:** The titer of injected AAV vector.

Vector	Titer (VG/ml)
AAV2.CMVeGFP	6.48 × 10^12^
AAV5.CMVeGFP	1.59 × 10^13^
AAV6.CMVeGFP	3.15 × 10^12^
AAV8.CMVeGFP	5.40 × 10^13^
AAV9.CMVeGFP	5.24 × 10^13^
AAVrh10.CMV.PI.eGFP.WPRE.bGH	4.16 × 10^12^

### *In vivo* intrathecal inoculation of AAV

All experimental procedures were approved by the Institutional Animal Care and Use Committee (IACUC) of Wake Forest School of Medicine, and performed in accordance with their guidelines and regulations. AAV-eGFP virus (10 µl) was injected intrathecally between the L4/5 vertebrae by percutaneous lumbar puncture into male hairless SCID congenic mice (6–8 weeks old; CB17.Cg-*Prkdc* ^*scid*^ *Hr* ^hr^/lcrCrl; Charles River Laboratories, Wilmington, MA), using a 30-gauge needle connected to a connecting joint (Eicom, cat #: JB-30, San Diego, CA) and a Hamilton syringe (Hamilton, Reno, NV). The appropriate insertion of the needle into the intrathecal space was confirmed by tail-flick prior to administration of virus into the subarachnoid space. Four weeks after the virus injection, mice were perfused with 4% paraformaldehyde (PFA); and the lumbar spinal cords, (L2-L4) DRGs, femurs, and hind paw skins were dissected and prepared for immunofluorescence assays.

### Tissue processing

The spinal cord, DRG, and paw skin sections were post-fixed with 4% PFA for 6 hours at 4 °C, and cryoprotected with 30% sucrose solution (in phosphate-buffered saline, PBS) for 48 hours at 4 °C. The tissues were then embedded in Tissue-Tek optimum cutting temperature (OCT) compound (Sakura finetek USA, Torrance, CA), and frozen on dry ice. Frozen spinal cord, DRG, and paw skin were thaw-mounted on Super Frost non-adhesion glass slides (Fisher Scientific, Hampton, NH) at 40 µm thickness, Super Frost non-adhesion glass slides coated with gelatin (Fisher Scientific) at 16 µm thickness, and gelatin-coated glass slides at 20 µm thickness, respectively.

The excess muscles were removed from the dissected femurs, using surgical scissors and a scalpel without disturbing the periosteum around the femur. The femurs were post-fixed with 4% PFA for 6 hours, and then decalcified with 10% ethylenediaminetetraacetic acid (EDTA, in pH 7.3 PBS), for 14 days (the decalcification solution was replaced on day 8) at 4 °C. After decalcification, the femurs were cryoprotected with 30% sucrose solution (in PBS) for 48 hours at 4 °C. The day before freezing on dry ice, the femurs were embedded in OCT compound and incubated overnight at 4 °C. The frozen femurs were thaw-mounted on gelatin-coated glass slides at 20 µm thickness.

### Immunofluorescence assays

Spinal cord was stained using the free-floating method, as previously described^21^, while DRG, bone, and paw skin were stained on the slide. After incubating with blocking buffer (3% Normal Donkey serum [Jackson ImmunoResearch, West Grove, PA], and 0.3% Triton X-100 [Sigma-Aldrich, St. Louis, MO] in PBS) for 1 hour at room temperature; spinal cord, DRG, bone, and paw skin sections were stained with chicken anti-GFP antibody (1:1000, Invitrogen, cat #: A10262, Carlsbad, CA) to enhance the eGFP signal. Bone and paw skin sections were co-stained with anti-PGP9.5 antibody (1:500; Proteintech, cat #: 14730-1-AP, Rosemont, IL). In some cases, spinal cord and DRG sections were co-stained with either mouse anti-NF200 antibody (1: 2,000, Abcam, cat #: ab134459, Cambridge, MA), rabbit anti-CGRP antibody (1:10,000, Sigma-Aldrich, cat #: C8198), or biotinylated IB4 antibody (1:2,500, Sigma-Aldrich, cat #: L2140). These primary antibodies were incubated overnight at 4 °C with gentle agitation. Thereafter, secondary antibodies (anti-rabbit CY3 [1:600, Jackson ImmunoResearch], anti-chicken CY2 [1:500, Jackson ImmunoResearch], or anti-streptavidin CY5 [1:400, Jackson ImmunoResearch]) were applied, based on primary antibodies used, for 2 hours at room temperature. DRG, bone, and paw skin sections were mounted in ProLong^TM^ Gold antifade mount (Fisher Scientific) with DAPI for nuclei labeling. Spinal cord sections were dehydrated using different gradients of ethanol (70, 90, and 100%, for 2 minutes each), cleared with xylene (twice for 2 minutes each), and coverslipped.

### Imaging and quantification

Immunofluorescence assays were performed using a Nikon Eclipse Ni fluorescent microscope system (Nikon, Tokyo, Japan) or Olympus FV1200 SPECTRAL Laser scanning confocal microscope (Waltham, MA). For DRG quantification, 4–5 DRG sections from each mouse (n = 3) were randomly selected, and GFP, NF200, CGRP, and IB4 positive DRG cells were counted, using an image analysis software, Nikon Elements V4.13 Basic Research. Data were expressed as percentage of the total number of DRG cells that expressed a given marker. For bone and paw skin quantification, 5–6 bone and 3–4 skin sections from each mouse (n = 3) were randomly selected, and GFP or PGP 9.5 positive area was determined within the same region of interest, using an image analysis software, Nikon Elements V4.13 Basic Research. The data were expressed as percentage of GFP positive area over PGP9.5 positive area.

### Statistical analysis

Numerical data are expressed as mean ± standard deviation. Statistical analysis was performed by unpaired two-tailed Student’s *t* test or one-way ANOVA with Tukey’s multiple comparisons, using the GraphPad Prism statistical program (GraphPad Software, San Diego, CA) with significance at *P* ≤ 0.05.
